# A Daily Breathing Practice Bolsters Girls’ Prosocial Behavior and Third and Fourth Graders’ Supportive Peer Relationships: A Randomized Controlled Trial

**DOI:** 10.1007/s12671-023-02158-9

**Published:** 2023-06-15

**Authors:** Maria von Salisch, Katharina Voltmer

**Affiliations:** Institute for Sustainability Education and Psychology, Leuphana University Lüneburg, Universitätsallee 1, D-21335 Lüneburg, Germany

**Keywords:** Breathing practice, Primary school, Children, Prosocial behavior, Supportive peer relationships, Gender differences

## Abstract

**Objectives:**

In order to promote mindfulness in primary school, the Breathing Break Intervention was developed. This collection of short daily breath-based mindfulness practices was introduced to 15 teachers who delivered them up to 3 times a day to their students.

**Method:**

In a randomized controlled trial, 146 third and fourth graders (49% female) either received the intervention (*n* = 81) or participated in the active wait list control group (*n* = 65). Students were asked to nominate prosocial peers and to report on supportive peer relationships in their classrooms before (pretest) and after (posttest) the 9 weeks of the Breathing Break Intervention, and in a follow-up 5 months later.

**Results:**

Mixed multilevel models indicated a group × sex × posttest interaction (*t*(211) = 2.64, *p* < 0.01) suggesting that girls in the intervention group were rated to be more prosocial by their peers at posttest than at pretest and than girls in the active control group when children’s age and parents’ education were accounted for. Supportive peer relationships in the active control group deteriorated between pretest and posttest, which occurred immediately before the second school lockdown due to the COVID-19 pandemic, whereas they remained the same in the intervention group (*t*(223) = 2.56, *p* < 0.05). Both effects were not maintained at follow-up, probably due to children’s irregular school attendance during the lockdown.

**Conclusions:**

Introducing a short daily breathing practice in primary school classrooms seems to be effective in maintaining supportive peer relationships and in stimulating girls’ prosocial behavior.

**Preregistration:**

The study was preregistered at aspredicted.org (#44925).

**Supplementary Information:**

The online version contains supplementary material available at 10.1007/s12671-023-02158-9.

For children, learning in school is a social affair, especially in primary school. Being included in social and learning activities (such as group work, projects, or sports teams) is crucial for school children’s well-being (Savahl et al., 2019), school engagement (Halliday et al., 2021), and last but not least academic achievement (Hattie & Yates, 2013). With a short daily mindfulness practice, our intervention aims to support children at the end of primary school to become more aware of their own experiences and those of others and to behave in a friendly and prosocial way. According to best evidence from recent meta-analyses, experiencing mindfulness enhanced children’s prosocial behavior, resilience, executive function, and attention, and decreased their anxiety (Felver et al., 2016; Phan et al., 2022), whereas supportive peer relationships in late childhood supported their emotion regulation, affective social cognition, social skills, and optimism (Mitic et al., 2021). Both mindfulness and positive peer relationships seem to contribute to the development of the “social brain” which may be critical for the maturation of the social-emotional and cognitive skills that may prevent some of the mental health problems (i.e., anxiety and depression) which are so prevalent in (early) adolescence (e.g., Wong et al., 2018).

According to the nationally representative German “Health Interview and Examination Survey for Children and Adolescents” (KiGGS) study, 18% of German primary school children were rated by their parents to have emotional problems (Hölling et al., 2014). Much of children’s emotional turmoil is caused or exacerbated by bullying and other aggressive or derogatory behavior by their classmates (e.g., Halliday et al., 2021). Since 2002, between 7.8 and 11% of the 11- to 15-year-olds in each of the representative samples of the “Health Behavior in School-aged Children” study reported that they had been bullied by peers (Fischer et al., 2020). In the representative World Vision Children’s Study, children from families of a lower socioeconomic status (SES) experienced marginalization more frequently (Andresen et al., 2018). International studies agree that a small but consistent number of students is bullied in the average classroom. Victimization by peers contributed to children’s anxiety, low school engagement, and low academic performance (Halliday et al., 2021). Feeling socially excluded in early adolescence explained how much of the gray matter remained in a distress-related brain region, whereas in typical development it tended to decrease over time (Raufelder et al., 2021). Building students’ social and emotional competence in the classroom is therefore of paramount importance.

According to Denham and Brown’s (2010) model of social-emotional learning, competent behavior is demonstrated by youths who have high self- and social-awareness, can regulate their emotions well, make responsible decisions, and exhibit prosocial behavior in the sense of cooperation and sharing as core relationship skills. Behaving in a prosocial way bolstered children’s acceptance by classmates and their academic success (Guo et al., 2018) as does social awareness (Voltmer & von Salisch, 2017). Prosocial behavior in the sense of intentional, voluntary, and sometimes altruistic helping behavior among the children is part of a positive learning culture in the classroom. In Jennings and Greenberg’s (2009) model, a prosocial classroom contributes to students’ social, emotional, and academic success, increases teachers’ enjoyment, and lowers their risk for burn-out.

Children’s prosocial behavior contributed to the development of supportive peer relationships (Mitic et al., 2021). Having high-quality relationships to classmates and not being ridiculed or excluded by their peers because of apparent flaws were core elements of a positive classroom climate (Jennings & Greenberg, 2009). A supportive social climate among peers contributed to students’ engagement and success in learning with a medium effect size of *d* = 0.53 (Hattie & Yates, 2013). According to another meta-analysis over 61 studies with 679 effect sizes and over 73,000 participants, classroom climate with high instructional and emotional teacher support and consistent classroom management contributed positively to youths’ social competence, academic achievement, and motivation, and negatively to their externalizing behavior and socioemotional distress (Wang et al., 2020). The socioemotional support dimension of classroom climate (which included peer relationships) was closely linked to children’s socioemotional distress. Students’ social well-being was also bolstered by the sense of community in the classroom, which in turn was influenced by peer relationships within the class (Capone et al., 2018). Being part of a positive learning community seems to fulfill children’s basic psychological need of relatedness (Deci & Ryan, 2008). Mindfulness exercises may help in building a supportive climate among classmates.

According to a well-known definition, mindfulness is “paying attention in a particular way: on purpose, in the present moment, non-judgmentally” (Kabat-Zinn, 1994, p. 4). A first element of mindfulness is the conscious awareness in the present moment of body signals, sensory perceptions, feelings, and thoughts. A second element is to accept them as present and to let them go. Children can learn to focus their attention on the present moment. Non-judgmental acceptance of their sensations helps them to let go of their judgements which may contribute to prosocial behavior. Frequent practice is important, because each practice tends to strengthen the neural circuits between the prefrontal cortex and the amygdala in children (Zelazo & Lyons, 2012). Several functional magnetic resonance imaging studies have demonstrated that the amygdala of adults who underwent Mindfulness Based Self-Regulation (MBSR) training for 8 weeks was activated less frequently. It was also more strongly connected to the prefrontal cortex, and it returned to baseline more quickly after an emotional stimulus, indicating improved emotion regulation (e.g., Gotink et al., 2016).

Prosocial behavior is targeted in many mindfulness programs for children. In the MindUP program, for example, prosocial behavior was promoted by a daily practice of mindful listening, by practicing mindful talking, and by acts of kindness towards classmates, family, and community members. After a 9-week MindUP intervention in Canada, fourth graders’ prosocial behavior increased in the eyes of their peers. That is, sharing and trustworthiness as well as helpfulness and perspective taking intensified, whereas prosocial behavior in the control group decreased on average (Schonert-Reichl et al., 2015). The meta-analysis by Klingbeil et al. (2017) over 12 intervention studies with 1105 participants confirmed that mindfulness interventions positively affected children’s social competence and prosocial behavior (Hedges’ *g* = 0.37, 95% CI [0.16, 0.57], *p* < 0.002). A recent study by Janz et al. (2019) in the early grades of primary school highlighted that the “Calm Space” mindfulness intervention improved the prosocial behavior of children in the intervention group (compared to the control group) when delivered by their teachers during regular lessons in the classroom.

Only a few studies have addressed the effects of mindfulness interventions on supportive peer relationships or social climate in the classroom. Lombas et al. (2019) presented a teacher training that enabled teachers to conduct mindfulness and “character strengthening” exercises with *N* = 524 adolescent students (*M* = 13.6 years). In the evaluation of this Spanish intervention with a pre-post comparison without a control group, students’ self-reports of self-esteem and life satisfaction increased, whereas their aggressiveness decreased. No effects were found, however, on the perceived social relationships among the adolescents. The study by Anheyer et al. (2021) with German grade schoolers found an improved social climate in the classroom after their mindfulness intervention. In both studies, a control group was missing, so that alternative explanations cannot be excluded. The present study aims to provide evidence for a mindfulness intervention promoting prosocial behavior and a supportive climate in the classroom in a randomized controlled trial (RCT) with an active control group (ACG).

Because mindfulness can be understood as a continuum of practices (Kropp & Sedlmeier, 2019; Lutz et al., 2015) or components (e.g., Singer & Engert, 2019) with differential effects, this study concentrated on practicing focused attention with a clearly defined object, i.e., children’s experience of breathing and observing sensory experiences within the body. These are basic techniques of “body centered meditation” in Matko and Sedlmeier’s (2019) empirical classification. The Breathing Break Intervention is a short, teacher-administered daily practice in the classroom. During the intervention, teachers invited the children in their classrooms to participate in 3- to 5-min practice sessions up to 3 times each school day. They chose from 15 exercises out of a manual (see appendix) depending on what was needed in the classroom because some exercises were designed to activate students whereas others intended to calm them down, and still others focused on their awareness of hands and feet. Teachers were free to discuss students’ experiences with them, but verbalizing inner experiences was not the main point of this intervention (Martens et al., 2020). Learning objectives of the exercises can be found in the appendix. Details on their delivery are provided in the “Method” section.

The Breathing Break Intervention was delivered to third and fourth graders because there is a considerable overproduction of synapses in the prefrontal cortex which seems to set the stage for advances in executive functions and their associated self-regulatory capacities during puberty (Zelazo & Carlson, 2012). Relatedly, most children in late childhood have acquired advanced levels of theory of mind which allow them to represent three different, but interrelated, mind sets at the same time (Osterhaus & Koerber, 2021). This facilitates acting in a prosocial way, even in more complex social situations. Learning about mindfulness also familiarizes children early on with methods of how to cope with the psychological effects of the pubertal changes in their bodies and minds. Mindfulness allows them to react with kindness to parallel changes in their peers, individually and in a classroom (Roeser & Pinela, 2014). This may prevent some of the mental health problems, such as anxiety or depression, which increase sharply in early adolescence and tend to persist. In fact, 50% of adults with psychiatric disorders experienced clinically impairing psychopathology before their fifteenth birthday (Kessler et al., 2005). Finally, targeting children in primary school entails reaching nearly all children of a birth cohort. It prepares them for the transition to secondary school in grade five, when children start to follow different tracks (i.e., different types of school) in the German stratified school system. Because the SES of children’s families seems to moderate the effects of mindfulness interventions (Lassander et al., 2022), their parents’ education was included as a background variable. The current study evaluated the effects of the teacher-led Breathing Break Intervention on children’s prosocial behavior and their perceptions of the social classroom climate in a RCT with an ACG and age, gender, and parental education as covariates. It aimed to answer the following question: What is the effect of the Breathing Break Intervention on supportive peer relationships and prosocial behavior (while controlling for the covariates)? It was expected that children in intervention group (IG) (1) will perceive peer relationships within their classrooms to be more supportive and (2) will be rated to act more prosocially after the intervention by their classmates (2a) and teachers (2b) when compared to their pretest values and to the ACG. This would be demonstrated in a significant interaction between group (i.e., IG vs. ACG) and time.

## Method

### Participants

Written invitations to receive the teacher training and to participate in the study were sent to 26 elementary schools in the city and the countryside surrounding a medium-sized city in Lower Saxony, Germany. Nine classroom teachers of five elementary schools agreed to participate, knowing that they could either be assigned to the intervention group (IG) or to the active waitlist control group (ACG). For the randomized controlled trial (RCT), randomization took place by drawing lots with participating classes from one school always assigned to either the IG or the ACG to avoid contamination. The participating classes included *n* = 154 children from *n* = 9 third- and fourth-grade classrooms. There were *n* = 8 children who were only nominated by their peers (because they had not been allowed to participate in the evaluation). These students were excluded from all analyses, because there was no additional data on them. Afterwards, the total sample size was *n* = 146 with *n* = 81 in the IG and *n* = 65 in the ACG, with some children missing at one or two measurement points. At the pretest (T1), direct data and information from teachers about children were collected for *n* = 140 children. At the posttest (T2) eight children (five in the ACG) dropped out of the sample, but five children who were not in the pretest joined the ACG. At the follow-up (T3), seven additional children dropped out (three in ACG) whereas one child who had not participated before and five children who had missed the posttest joined the sample (all ACG). Thus, sample sizes for pretest, posttest, and follow-up were *n* = 140, *n* = 137, and *n* = 136, respectively. The reasons for missing measurements and drop-out were illness, moving to another city, and refusal to participate. Of the pretest sample, 94% participated in the posttest and 93% in the follow-up data collection.

At the pretest, *n* = 81 children were in the IG and *n* = 59 children were in the ACG. The distribution of children’s sex (49% female) did not differ between groups (*Χ*^2^(1) = 1.73, *p* = 0.188). The age of the children in the ACG (*M* = 8.76 years, *SD* = 0.80, range = 8–11) was higher than in the IG (*M* = 8.31 years, *SD* = 0.56, range = 7–10; *t*(98) = 3.76, *p* < 0.001) because half of the children in the ACG were in fourth grade, whereas all children of the IG attended third grade. Eight children had special educational needs (learning, *n* = 4; physical development, *n* = 1; emotional development, *n* = 3, chronic illness, *n* = 1) with one child being diagnosed with two special educational needs. Parents’ questionnaires were used to record children’s bilingualism and socioeconomic background in terms of the highest educational attainment within the family. In the pretest sample, 27 children (19%) were dual language learners (DLL). With 27% in the ACG versus 14% in the IG, the proportion of DLLs was somewhat higher in the ACG (*Χ*^2^(1) = 3.20, *p* = 0.074), but this was not significant. Parents’ highest educational attainment could be calculated for *n* = 134 children (90%) and is shown in Table 1. Overall, in *n* = 104 families (78%), at least one parent had a vocational qualification (vocational training or apprenticeship, technical college degree, or university degree). This proportion did not differ significantly between the ACG and the IG (*Χ*^2^(1) = 1.32, *p* = 0.251).

**Table 1:** Distribution of the highest educational attainment in the families of the children

	*n*	%
No graduation	5	4
Secondary school diploma^1^	18	13
A-levels	7	5
Vocational training	24	18
Technical college degree	12	9
University degree	68	51
Total	134	100

^1^And comparable degrees

At pretest, most children (73%) reported that they had had no prior experience with breathing exercises. A proportion of 9% engaged in breathing exercises for relaxation once a month, 10% once a week, and 8% more frequently. Overall, children in the ACG reported doing breathing exercises on their own more often than children in the IG (*Χ*^2^(1) = 6.02, *p* = 0.014).

A total of *n* = 15 female teachers filled in the paper-pencil questionnaires about themselves at pretest. Five IG teachers and four ACG teachers were classroom teachers who provided information about each child in their class. The remaining six teachers (who were all but one in the IG) agreed to lead the Breathing Breaks or the activity in the control condition. They only filled in the questionnaires about themselves. Table 2 shows that teachers’ professional experience, their mindfulness practice, and their self-reported mindfulness on the Five Facet Mindfulness Questionnaire (FFMQ; Baer et al., 2008; Gu et al., 2016; Michalak et al., 2016) did not differ between IG and ACG teachers. Teachers in the ACG had taught the children in their classes for longer periods before participating in the study than teachers in the IG, probably because half of the children were already in fourth grade. None of the classrooms had participated in a mindfulness project before.

**Table 2:** Teachers’ characteristics in the intervention group and the active wait list control group

	IG (*n* = 11)	ACG (*n* = 4)	Test
Professional experience			Fisher’s exact: *p* = 0.585
0–2 years	2 (18%)	0 (0%)	
6–10 years	2 (18%)	1 (25%)	
11–20 years	4 (37%)	3 (75%)	
> 20 years	3 (27%)	0 (0%)	
Teaching the class			Fisher’s exact: *p* = 0.030
0 years	7 (64%)	0 (0%)	
1 year	1 (9%)	0 (0%)	
2 years	2 (18%)	1 (25%)	
3 years	1 (9%)	3 (75%)	
Experience with mindfulness practice			Fisher’s exact: *p* = 0.451
Never	6 (55%)	1 (25%)	
<1×/month	1 (9%)	2 (50%)	
1–6×/month	3 (27%)	1 (25%)	
1×/week or more	1 (9%)	0 (0%)	
FFMQ (means and SD)			Kruskal-Wallis rank
Acting with awareness	10.91 (2.34)	11.00 (2.58)	*Χ*^2^(1) = 0.00, *p* = 0.947
Describing	13.00 (1.63)	13.00 (1.50)	*Χ*^2^(1) = 0.00, *p* = 0.947
Observing^1^	11.82 (2.44)	13.00 (1.73)	*Χ*^2^(1) = 0.31, *p* = 0.578
Nonjudging	12.91 (1.81)	11.75 (2.22)	*Χ*^2^(1) = 0.86, *p* = 0.353
Nonreacting	10.55 (1.37)	9.25 (1.26)	*Χ*^2^(1) = 2.38, *p* = 0.123
Overall^1^	58.82 (5.67)	59.67 (3.51)	*Χ*^2^(1) = 0.02, *p* = 0.876

^1^*n* = 14, because of missing data. *IG*, intervention group; *ACG*, active control group; *FFMQ*, Five Facet Mindfulness Questionnaire

### Procedure

After obtaining permission from the Ethics Review Board of our university (on May 6, 2020), pretest data were collected in September 2020 and posttest data in December 2020 just before the second COVID-19 pandemic-related lockdown in Germany. The follow-up was conducted in May 2021 when schools had just reopened. Data were collected by means of tablet computers. Items and instructions were read to the children by a test manager in the front of the classroom. The children could read along silently for themselves. Three to four additional trained undergraduate research assistants supported children with technical difficulties or insufficient computer skills in answering on the tablets. Because of the COVID-19 pandemic, children’s classes were split up into two groups for several months in the spring of 2021, with each group being in school on 2 or 3 days a week. During the follow-up data collection, classrooms were also divided into two groups each. Our survey lasted three to four periods at each measurement point and was interrupted by the usual breaks between the lessons. Children took part in the evaluation study only after they had brought the written consent of a parent or guardian. Children’s participation was voluntary. Children who were not allowed or did not want to participate were either supervised in another room or quietly occupied themselves with materials provided by the teacher. After completing the evaluation study, all children received a small gift. Teachers filled in the rating scales about each child of their class and paper-pencil questionnaires about themselves. Together with their consent form, parents completed a short questionnaire about their family situation, the language(s) spoken at home, and their educational and professional background.

In June 2020, the nine teachers of the IG received the Breathing Break curriculum and underwent a short mindfulness training with an external, certified MBSR trainer. These were the five classroom teachers and one additional teacher for all but one classroom. In three sessions and a day of mindfulness (15 hr in total), they were introduced to the concept of mindfulness, practiced key mindfulness exercises, and were advised on how to lead the breathing exercises for the children in their classes. Before starting the Breathing Break Intervention, IG teachers met with the researchers to settle the last questions before the pretest. Teachers’ breathing practice with the children was supervised by another certified MBSR teacher with ample experience of teaching mindfulness in primary school.

The Breathing Break Intervention was conducted between pretest and posttest for about 10 weeks in each of the five intervention classes. Teachers were asked to conduct the Breathing Breaks with their class up to three times a day on every school day, and to record which of the 15 exercises they had chosen in an implementation calendar (see below). A short description of the learning objectives for each group of exercises is provided in the appendix. At the posttest, IG teachers and children were asked whether they wanted to continue the Breathing Breaks in school after the Christmas holidays. However, due to the Germany-wide pandemic-related lockdown between December 2020 and March 2021, most children were taught in distance classes, so that it was impossible to continue with the Breathing Breaks in school.

ACG teachers were asked to include coloring mandalas in their lessons up to three times on every school day for the same 10 weeks as the IG. They were also asked to record the number of coloring breaks they had conducted.

### Measures

#### Peer rating of Prosocial Behavior

Based on the German version of the Adjustment Scales for Sociometric Evaluation of Secondary-School Students (Pössel et al., 2005), the prosocial behavior of each child was recorded at all measurement points by asking all participating children two questions: “Who in your class can work well in groups?” and “Who in your class likes to share?” The children were asked to write up to three names of their classmates into their tablets. The research assistants made sure that the children did not chat with each other or look at what other children were writing while answering these questions. Afterwards, children’s names were replaced by their codes. The number of ratings on the two questions correlated highly with *r* = 0.55 (pretest), *r* = 0.60 (posttest) and *r* = 0.64 (follow-up). A sum of the two scores was calculated and for each child’s code the number of nominations received was tallied and divided by the total number of nominations in his or her classroom. Children’s nomination rates varied between 0 and 23% at the pretest. Children with higher nomination rates were considered to be more prosocial. Cronbach’s *α* for the 2-item scale was acceptable with 0.71. McDonald’s *ω* could not be computed because of the limited number of items. Test-retest reliability of the peer nominations was high with *r*_pre-post_ = 0.65, *r*_pre-follow-up_ = 0.61, and *r*_post-follow-up_ = 0.73.

#### Classroom climate

The subscale “classroom climate” of the German questionnaire to assess the emotional and social school experiences of third and fourth grade school children (FEESS 3-4; Rauer & Schuck, 2003) was used at all measurement points to assess each child’s perception of the social climate among peers in the classroom. Classroom climate is defined in the manual as the “extent to which the children in the class interact in a socially appropriate and friendly manner and have a good relationship with each other” (Rauer & Schuck, 2003, p. 9, authors’ translation). The subscale includes 11 items that are scored on a Likert-type scale (0 = *not at all true*, 1 = *hardly true*, 2 = *somewhat true*, 3 = *exactly true*). Six items were reverse scored. Raw scores could thus range from 0 to 33 with higher scores indicating a more supportive classroom climate. An example item is “We make fun of some children” (reverse scored). Internal consistency was acceptable with *α* = 0.72 and *ω* = 0.72 at pretest. Test-retest reliability was moderate with *r*_pre-post_ = 0.51, *r*_pre-follow-up_ = 0.40, and *r*_post-follow-up_ = 0.57.

#### Children’s Acceptance

IG children’s acceptance of the Breathing Breaks was obtained by the “Three-finger-evaluation.” In this feedback sheet, children wrote down “what I liked” (finger 1), “what I did not like” (finger 2), and “what could be done better” (finger 3) about the Breathing Breaks about 2 weeks after the start of the intervention and at posttest. Children’s feedbacks on what they liked were transcribed and later categorized as “Movement,” “Relaxation, quietness, calm,” “Calming breathing exercises,” “Activating breathing exercises,” “Arrangement,” “Everything,” and “Further comments” (Möller, 2021). At posttest, additional feedback for all Breathing Breaks was obtained from the IG children in a short “Consumer satisfaction questionnaire.” In it, children were asked to indicate (among other things) whether they wanted to continue the Breathing Breaks in class in a “yes”-“no” question. At follow-up, children were asked how frequently they had performed the Breathing Breaks “on their own,” i.e., outside of class, since the posttest about 5 months earlier.

#### Teacher Rating of Prosocial Behavior

Teachers filled in the Strengths and Difficulties Questionnaire (SDQ; Goodman, 1997; Petermann et al., 2010) for each child in their classroom at all three measurement points. The prosocial behavior subscale of the SDQ contains five items with an answer format of *not true*, *somewhat true*, and *certainly true* with corresponding scores of 0, 1, and 2. None of the items of this subscale was reverse scored. Item scores were added up, so that total scores could range between 0 and 10 with higher scores indicating more consistent prosocial behavior. An example item is “Helpful if someone is hurt, upset, or feeling ill.” Internal consistency was good with *α* = 0.89 and *ω* = 0.89 for this subscale at the pretest. Test-retest reliability was high with *r*_pre-post_ = 0.78, *r*_pre-follow-up_ = 0.74, and *r*_post-follow-up_ = 0.83.

#### Teacher Report on Mindfulness

At T1, teachers filled in the German translation of the short form of the Five Facet Mindfulness Questionnaire (FFMQ; Baer et al., 2008; Gu et al., 2016; Michalak et al., 2016) which addressed the following facets of mindfulness: Acting with Awareness, Describing, Observing, Nonjudging, and Nonreactivity to Inner Experience. The 15 items of the FFMQ are scored on a Likert-type scale (1 = *never or very rarely true*, 2 = *rarely true*, 3 = *sometimes true*, 4 = *often true*, 5 = *very often or always true*). Seven items were reverse scored. Raw scores could thus range from 1 to 5 for each subscale with higher scores indicating more mindfulness. A total score for mindfulness was formed by summing the raw scores. A sample item of the Describing scale is “I’m good at finding words to describe my feelings.” Internal consistency for the total score was barely acceptable with *α* = 0.66 at pretest. Because of items that correlated negatively with the total scale, McDonald’s *ω* could not be computed. Test-retest reliability was moderate with *r*_pre-post_ = 0.50, *r*_pre-follow-up_ = 0.19, and *r*_post-follow-up_ = 0.11.

#### Implementation Calendar

IG teachers filled in a calendar for each school day during the intervention. In the calendar, they wrote down the exercises they had performed with the class and comments on how it went (if they liked). Because the exact number of intervention days varied between classrooms (because of scheduling problems), the number of maximally possible Breathing Breaks (3 *number of days) varied between 148 and 164. In order to obtain a comparable measure across classrooms, the proportion of actually delivered Breathing Breaks (between 65 and 149) out of the maximally possible Breathing Breaks was calculated for each classroom. In sum, teachers led between 41 and 91% of the maximally possible Breathing Breaks. It happened that, for example, due to the absence of trained teachers, in some classes, Breathing Breaks were not carried out at least once a day. ACG teachers were asked to record the number of coloring breaks they did every day. It ranged from one to three breaks per day. On some days, teachers could not do any coloring breaks, because of illness, project days, or COVID-19 quarantine.

#### Teachers’ Acceptance

Teachers were asked at the follow-up to report in retrospect how many Breathing Breaks they had led with their class since the posttest about 5 months earlier, whether they benefited from the Breathing Breaks themselves, whether they noticed a change in their relationship with the children, whether they would like to do the exercises in the future and with other classes, whether they would be interested in introducing the Breathing Breaks to their colleagues, and whether they found the Breathing Breaks generally helpful.

### Data Analyses

All statistical analyses were conducted with the software R (R Core Team, 2020). Before the main analyses, data were screened for outliers and multicollinearity. Effects of the Breathing Break Intervention on children’s prosocial behavior and classroom climate were tested by means of multilevel linear mixed model analyses, in which the repeated measures of the outcome variables were nested within subjects and subjects were nested within classrooms.

## Results

Boxplots were used to screen all relevant variables for outliers. For the peer ratings of prosocial behavior at all measurement points, and for classroom climate at follow-up, potential outliers were detected. The Rosner Test (Rosner, 1983) was used to check whether these potential outliers differed significantly from the other values. One subject was identified to have received an extremely high number of peer nominations for prosocial behavior at all measurement points. However, after re-examining the raw data and concluding that these values were plausible and a product of natural variation, these outliers were not excluded from the analyses.

Descriptive statistics and bivariate correlations between all variables are displayed in Table 3. Because children’s sex correlated significantly with almost all indicators of prosocial behavior, it was included in the main analyses. Because children’s age and their parents’ level of education correlated significantly with some of the outcome variables, they were also included in the main analyses. Parents’ educational attainment was combined into a dichotomous variable for the main analyses (vocational qualification vs. no vocational qualification within the family) in order to obtain comparison groups of sufficient size.

**Table 3:** Descriptive statistics and bivariate Bonferroni-corrected Spearman correlations of children’s characteristics and relevant variables

		1	2	3	4	5	6	7	8	9	10	11	Mean (*SD*)	Range	Skew	Kurtosis
1	Age in months												106.69 (7.88)	95–134	0.97	3.86
2	Sex (0 = male)	−0.10											-	-	-	-
3	Parent education	−0.22	−0.07										-	-	-	-
4	Pre SDQ prosocial	0.09	0.34	−0.08									7.37 (2.29)	2–10	−0.21	1.82
5	Post SDQ prosocial	0.15	0.28	−0.09	0.78								7.72 (2.15)	1–10	−0.64	2.55
6	Follow-up SDQ prosocial	0.20	0.31	−0.08	0.74	0.83							7.67 (2.17)	2–10	−0.40	1.84
7	Pre peer prosocial	−0.18	0.21	0.18	0.34	0.36	0.40						0.06 (0.05)	0.00–0.23	1.11	4.11
8	Post peer prosocial	−0.09	0.34	0.27	0.31	0.33	0.32	0.65					0.06 (0.05)	0.00–0.23	1.07	4.08
9	Follow-up peer prosocial	−0.14	0.22	0.19	0.31	0.35	0.34	0.61	0.73				0.06 (0.05)	0.00–0.30	1.25	6.21
10	Pre class. climate	−0.17	0.20	0.09	0.15	0.20	0.21	0.17	0.11	0.06			24.72 (5.12)	11–33	−0.45	2.68
11	Post class. climate	−0.24	−0.06	−0.03	0.03	0.08	0.10	0.13	0.05	0.07	0.51		23.75 (6.09)	5–33	−0.50	2.58
12	Follow-up class. climate	−0.15	−0.08	−0.03	0.10	0.10	0.07	0.12	0.09	0.08	0.40	0.57	24.72 (5.25)	8–33	−0.51	2.93

Correlations > |0.17| are significant at *p* < 0.05. *SDQ*, Strengths and Difficulties Questionnaire; *Pre*, pretest; *Post*, posttest

Individual children’s perceptions of the social climate among peers in their classroom were not significantly associated with teacher or peer ratings of prosocial behavior at the same measurement point. Children who perceived the social climate to be more supportive at the pretest were rated to be more prosocial by their teachers at the posttest and at the follow-up. However, these were small correlations with little practical relevance. Therefore, children’s reports of a supportive classroom climate and their ratings on their prosocial behaviors were considered to be independent outcome variables.

### Effects on Classroom Climate

**Fig. 1:**
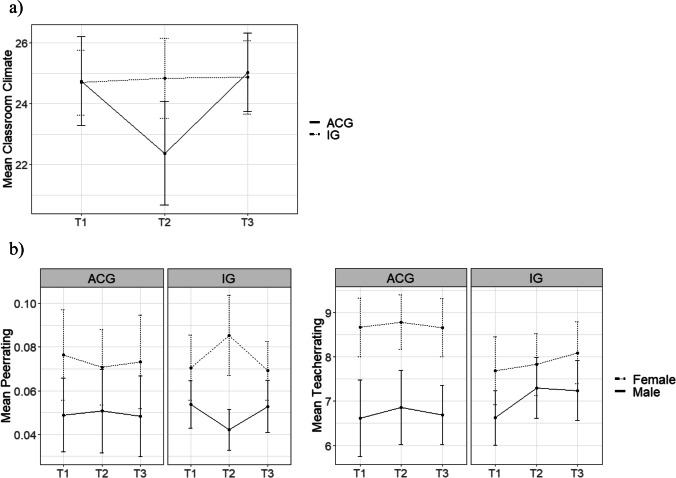
Development of the mean ratings (with 95% confidence intervals) of ***a*** a supportive classroom climate and ***b*** prosocial behavior over three measurement points; *ACG*, active control group; *IG*, intervention group

To examine the effect of the Breathing Break Intervention on classroom climate, a multilevel model with an interaction between binary indicators for measurements at pretest, posttest, and follow-up and group (IG vs. ACG) was fitted, which used the pretest measurement and the ACG as references and included child sex, age, and highest educational attainment within the family as control variables. Results of the model are displayed in Table 4. A significant main effect of children’s age on mean classroom climate was found, which indicated that older children perceived the classroom climate among peers to be less supportive. Furthermore, the model showed a significant interaction effect between posttest and group. As can be observed in Fig. 1a, IG children’s perceptions of the social climate in their classroom remained at the same mean level between pretest and posttest while those of ACG children grew more negative. No significant interaction effect was found between group and follow-up.

**Table 4:** Linear mixed model on the effect of the Breathing Break Intervention and time on children’s perceptions of a supportive classroom climate

	*β*	SE	df	*t*	
Intercept	26.93	1.48	223	18.23	***
Posttest	−0.21	0.82	223	−3.05	**
Follow-up	0.03	0.81	223	0.55	
Group (0 = *ACG*)	−0.04	1.53	7	−0.32	
Sex (0 = male)	0.02	0.74	123	0.26	
Parent education (0 = no vocational qualification)	0.07	0.92	123	1.05	
Age	−0.11	0.01	223	−2.26	*
Group * posttest	0.19	1.05	223	2.56	*
Group * follow-up	−0.01	1.05	223	−0.17	

***p < 0.001; **p < 0.01; *p < 0.05; ˙*p* < 0.10; *ACG*, active control group; *n* = 134

### Effects on Prosocial Behavior

To examine the effect of the Breathing Break Intervention on children’s prosocial behavior, two mixed models with triple interactions between the binary indicator variables for the pretest, posttest, and follow-up measurements, group (IG vs. ACG), and sex of the children were fitted. Again, the pretest measurement for prosocial behavior and the ACG were the reference groups, and family education and child age and sex were the control variables. The first model used the peer nominations and the second model the teacher ratings of prosocial behavior for each child.

The first model, which is displayed in Table 5, showed a significant triple interaction effect between time, group, and sex, indicating that at the posttest after the Breathing Break Intervention, girls of the IG were nominated by their classmates to be more prosocial than girls of the ACG and than at the pretest before the intervention (Fig. 1b). Again, no significant interaction effects were found between groups and changes between pretest and follow-up. Besides, the model showed a significant main effect of sex with girls receiving more nominations for being prosocial than boys from their classmates. Children with at least one parent with a vocational qualification were nominated to be more prosocial than children whose parents did not have a vocational qualification.

**Table 5:** Linear mixed model on the effect of the Breathing Break Intervention and time on peer nominations of children’s prosocial behavior

	*β*	*SE*	*df*	*t*	
Intercept	0.05	0.01	211	5.62	***
Posttest	0.00	0.01	211	0.41	
Follow-up	0.01	0.01	211	0.07	
Group (0 = ACG)	0.03	0.01	7	0.23	
Sex (0 = male)	0.32	0.01	122	2.53	*
Parent education (0 = no vocational qualification)	0.16	0.01	122	2.16	*
Age	−0.02	0.00	211	−0.41	
Group * posttest	−0.11	0.01	211	−1.30	†
Group * follow-up	0.02	0.01	211	0.20	
Sex * posttest	−0.07	0.01	211	−0.80	
Sex * follow-up	−0.03	0.01	211	−0.35	
Group * sex	−0.15	0.01	122	−1.02	
Group * sex * posttest	0.23	0.02	211	2.64	**
Group * sex * follow-up	0.03	0.02	211	0.40	

***p < 0.001; **p < 0.01; *p < 0.05; †*p* < 0.10; *ACG*, active control group; *n* = 134

Teacher ratings of children’s prosocial behavior did not show any significant interaction effects but only a main effect of child sex: Again, girls were rated to behave more prosocially than boys. Results are displayed in Table 6.

**Table 6:** Linear mixed model on the effect of the Breathing Break Intervention and time on teacher ratings of children’s prosocial behavior

	*β*	*SE*	*df*	*t*	
Intercept	6.68	0.69	226	9.74	***
Posttest	0.09	0.32	226	1.35	
Follow-up	0.05	0.31	226	0.71	
Group (0 = ACG)	−0.00	0.83	7	−0.01	
Sex (0 = male)	0.49	0.51	122	4.28	***
Parent education (0 = no vocational qualification)	0.00	0.38	122	−0.01	
Age	−0.01	0.00	226	−0.22	
Group * posttest	0.05	0.40	226	0.76	
Group * follow-up	0.09	0.39	226	1.24	
Sex * posttest	−0.04	0.43	226	−0.57	
Sex * follow-up	−0.07	0.42	226	−1.01	
Group * sex	−0.17	0.67	122	−1.34	
Group * sex * posttest	−0.06	0.56	226	−0.91	
Group * sex * follow-up	−0.01	0.56	226	−0.09	

***p < 0.001; **p < 0.01; *p < 0.05; †*p* < 0.10; *ACG*, active control group; *n* = 134

## Discussion

This RCT confirmed positive effects of the daily Breathing Break Intervention in third and fourth grade classrooms on peer nominations of girls’ prosocial behavior and on children’s perceptions of a supportive classroom climate at posttest in comparison to the pretest and to an active control group (ACG) when relevant controls (i.e., child age and parent education) were in place. This result underlines recent findings that school-based mindfulness practices tend to promote prosocial behavior in childhood (e.g., Janz et al., 2019; Schonert-Reichl et al., 2015) which were summarized in the meta-analyses by Klingbeil et al. (2017) and by Phan et al. (2022). One potential mechanism for the effect on prosocial behavior is children’s increased social awareness because the accurate and empathic perception of peers’ emotions is a prerequisite for helping them (Imuta et al., 2016).

In the present study, ratings of students’ prosocial behavior by peers and teachers correlated at a moderate level (0.31 < *r* < 0.40), because peer and teacher measures captured somewhat different aspects of prosocial behavior. The fact that the triple interaction of group, sex, and posttest was significant for the peer rating and not for the teacher rating of prosocial behavior suggests that the effects of the Breathing Break Intervention can be observed more easily at the concrete, observable level of behavior (i.e., sharing and working in groups, which was obtained in the peer nominations) than on the more abstract level of being helpful or considerate, which was asked in the teacher ratings. This is in line with the results of Schonert-Reichl et al. (2015) who also found large effects of their mindfulness intervention on peer ratings of sharing. However, Viglas and Perlman (2018) detected a positive effect of their mindfulness intervention in kindergarten on children’s teacher-rated prosocial behavior. Future studies should examine the effects of mindfulness-based interventions on different kinds of prosocial behavior in different age groups.

The increase of prosocial behavior on the part of the female participants in the IG may result from girls showing more acceptance of and being more engaged in the breathing exercises than boys. Whereas most children in the IG (between 91% after 2 weeks and 85% at posttest) wrote down positive experiences with the Breathing Break in the Three Finger Evaluation, girls seemed to appreciate the practices more fully than their male classmates. Whereas 35% of the girls valued the relaxing and quieting effect of the Breathing Breaks, only 19% of the boys did so (*t*(81) = −2.25, *p* = 0.03). In addition, more girls (13%) than boys (7%) wrote down positive experiences with activating breathing exercises, such as Rainbow Breathing (*t*(81) = −2.18, *p* = 0.04). However, as Bluth et al. (2017) pointed out, there is an urgent need for research to examine sex differences in response to mindfulness-based interventions in childhood and adolescence because of differences in their trajectories of emotional and cognitive development. In their study with *n* = 15 adolescents, female participants were more engaged in completing home practice and in class discussions and reported less stress after the intervention than male participants (Bluth et al., 2017). In a meditation study with *n* = 100 11- and 12-year-olds, Kang et al. (2018) corroborated gender differences in the sense that the affect of females in the intervention group improved when compared to females in the control group. An increase in self-reported self-compassion (which was associated with an improvement in affect) was observed only in girls, and not in boys. The question of whether boys and girls might benefit from slightly different types of mindfulness-based exercises (active vs. passive or activating vs. calming) also arose in their study and was further discussed by Rojiani et al. (2017) and Helminen et al. (2023). Qualitative research could be used to shed more light on these questions.

The present RCT extends the positive results of the Breathing Breaks from the level of individual students’ prosocial behavior to a supportive social climate at the level of the classroom. This effect dovetails with recent evidence (Kuyken et al., 2022; Meyer & Eklund, 2020) which was summarized in the meta-analysis by Dai et al. (2022). Peer relationships in the IG did not deteriorate like those in the ACG between pretest and posttest in September and December 2020. This can be explained by the strains brought about by the quickly rising incidence rates of the COVID-19 pandemic in Germany (Statista, 2021). During the intervention period, an entire class of the ACG was sent into quarantine for a number of days because one student was positively tested for the coronavirus. Other classes of the ACG were affected when teachers or classes from the same grade were quarantined. In all classrooms, the social climate was affected by school rules on social distancing among the children. In the IG, the deterioration of the climate was possibly ameliorated by performing the breathing exercises together. Thus, the Breathing Breaks may have acted as a team-building measure during a challenging period of time. The return to a more supportive classroom climate in the ACG at follow-up in May 2021 may be related to the mandatory COVID-19 testing in schools (since April 2021), which may have led to a more carefree (physical) contact among the children.

Although a healthy classroom climate theoretically contributes to children’s social and emotional wellbeing (e.g., Jennings & Greenberg, 2009), children’s perceptions of supportive peer relationships within their classrooms were statistically independent of their individual prosocial behavior. Those children who received many nominations for prosocial behavior did not perceive the social climate in their classrooms to be any better (or worse) than their less prosocial classmates. Because all participants nominated three prosocial children in this procedure, it was methodologically impossible to discover an association between the number of prosocial children (or the overall intensity of the prosocial behavior) in a classroom and the social climate in it. In the present study, supportive peer relationships did not seem to depend on individual, particularly prosocial children, but on the interactions of all classroom members.

Breathing Breaks acquaint primary school children with a daily breathing practice which can give some of them a respite in an often busy and sometimes hectic school day, as our student acceptance data (see above) suggest. Breathing Break is a suitable introduction to mindfulness for this pre-adolescent age-group, because the meditation focuses on the concrete object of breathing or body sensations and does not require extensive self-observation or verbalization of inner experiences (Lutz et al., 2015). Many other mindfulness-based intervention programs in schools also include breathing exercises as one aspect of mindfulness (e.g., McKeering & Hwang, 2019; Sapthiang et al., 2019). As a side effect, teachers may take part in the Breathing Breaks themselves — provided that the acceptance by their students is high, and they feel committed to the exercises. Teachers also appreciated taking a deep breath and spending a mindful moment while the class was still.

School is a suitable place for mindfulness interventions because all children are reached and because the breathing practice can be made into a ritual which promotes feelings of relatedness (Deci & Ryan, 2008) or cohesion within the class (Meyer & Eklund, 2020). Although mixed effects were obtained for having schoolteachers deliver lessons in mindfulness (e.g., Dai et al., 2022), training teachers is cost-effective, because they can use their mindfulness training for many years in future classrooms (e.g., Tarrasch & Berger, 2022). Many teachers can deliver Breathing Breaks with little extra training because the breathing exercises do not usually entail discussions of individual student’s feelings (inquiry), whereas giving instructions to students is part of their professional training. School is set up for daily breathing practice over longer periods of time which is necessary to strengthen neural pathways (Zelazo & Lyons, 2012). Quach et al. (2017) showed that home practice in mindfulness-based interventions with adolescents is implemented infrequently and inconsistently and that it is not effective in reducing perceived stress or anxiety symptoms. The review by Lloyd et al. (2018) corroborated that only half of the studies included found a significant effect of home practice on the outcomes of the mindfulness-based interventions. Regular practice increases the chances of achieving effects (Fredrickson et al., 2017; Zelazo & Lyons, 2012), provided that teachers succeed in accommodating individual children who do not feel comfortable with the breathing exercises with alternative forms of practice (e.g., Treleaven, 2018).

Contrary to expectations, the effects of the Breathing Break Intervention on children’s prosocial and supportive classroom behavior were not confirmed at the follow-up about 5 months after the end of the intervention. This may be explained by the closure of all schools and the provision of distance learning between December 2020 and April 2021 because of the COVID-19 pandemic. A proportion of 42% of the children in the IG rated the Breathing Breaks as “mostly good” or “always good” and 26% reported doing the exercises at home at least several times in a month at posttest. Teachers were encouraged to continue the Breathing Breaks after the intervention and over half of the children (53%) wanted to continue the Breathing Breaks in class at posttest. Although schools closed right after the posttest, 18% of the children in the IG continued to do the breathing exercises on their own “once a week” or “several times a week.” But the vast majority practiced the Breathing Breaks rarely or not at all during the following months. When schools reopened, most classrooms were split up into two groups of children with different schedules of attendance. Because there was much social turmoil and much pressure to “catch up” on lessons, few classroom teachers continued with the Breathing Breaks. In fact, 25% of the IG teachers reported that they had not led any Breathing Breaks between posttest and follow-up. Fifty percent reported that they had delivered Breathing Breaks approximately once a month and 12% each reported performing them once a week and more than once a week respectively. Because mindfulness interventions tend to be more effective when exercises are performed more frequently (e.g., Fredrickson et al., 2017; Zelazo & Lyons, 2012), the infrequent or unsteady performance of Breathing Breaks between posttest and follow-up in class may be a reason why the scores in the IG did not increase further. Other reasons may have to do with the social turmoil which was created by splitting up the classrooms into groups or with teachers’ emotional exhaustion because of the pandemic (e.g., Chan et al., 2021).

### Limitations and Future Directions

Strengths of the current study include the fact that the somewhat vague concept of mindfulness was decomposed into a clearly defined set of 15 exercises on breathing and body awareness, which were easy to understand and simple to perform for primary school children and which were easy to lead for their teachers. Intervention effects on classroom climate and girls’ prosocial behavior were obtained even though ACG children performed breathing exercises on their own more often than IG children at pretest. Methodological strengths include realizing a RCT and accounting for the nested structure of the data, as well as probing the fidelity of the intervention. Furthermore, the effects of the teacher-led Breathing Breaks could be demonstrated at the level of the children and at the level of the classroom. Limitations include the sample of children from one region of Germany, the increase in prosocial behavior only in girls, the lack of longitudinal effects due to the COVID-19 pandemic, and the lack of measuring the quality of the intervention by means of classroom observations for financial reasons. Because observation data are lacking, it cannot be ruled out that the self-reports and third-party reports were affected by common method bias. The large variances around the group means and the overlaps between the IG and the ACG suggest that additional variables need to be considered, which may moderate the results, such as dose effects, effects of children’s attention or mindfulness, or effects on children with and without disabilities in mainstreamed classrooms. Additional factors to be considered may be teachers’ appreciation of the Breathing Break and their own mindfulness practice because this may influence their way of leading the practice and engaging with the children in general. Future studies with larger and more varied samples should replicate our results and include possible moderators and mediators.

This study demonstrated that implementing regular mindful breathing exercises can improve primary school girls’ prosocial behavior and has the potential to stabilize a supportive classroom climate among peers during challenging times, such as social distancing. Being on the receiving end of their peers’ supportive behavior may convince children (and teachers) that it is a good idea to adopt Breathing Breaks as a classroom ritual for longer periods of time. Whether the effects of this breath-based mindfulness intervention are moderated by children’s acceptance of the Breathing Breaks (or the lack thereof) is a question for further research.

## Supplementary Information

ESM 1 Appendix (DOCX 18 kb)

## Data Availability

Data will be made available by the authors upon request.
