# Supplementary material for: A Daily Breathing Practice Bolsters Girls’ Prosocial Behavior and Third and Fourth Graders’ Supportive Peer Relationships: A Randomized Controlled Trial
Source: Mindfulness (N Y). 2023 Jun 15:1–14. Online ahead of print. doi: 10.1007/s12671-023-02158-9 (PMC10267544; doi:10.1007/s12671-023-02158-9)
Supplement: Supplementary file 1 — Appendix (DOCX 18 kb) [file 12671_2023_2158_MOESM1_ESM.docx]

# The exercises of the Breathing Break Intervention (BBI)

| **Main parts** | **Learning objectives:  Students** | **Name of exercise** | **Author** |
| --- | --- | --- | --- |
| **Intro-duction** | - …are invited to try out mindfulness positions while sitting or standing. | 1. king and queen posture | BBI |
| **Activating exercises** | - ...learn to feel their body.  - ...experience how it feels to be in contact with their breath.  - ...get to know their breath better.  - ...learn to talk about what is happening in their body and their breath.  - ...experience and allow a moment of silence. | 2. breathing probe | BBI |
|  |  | 3. rainbow breathing | W. Kinder |
|  |  | 4. switching on and off | W. Kinder |
|  |  | 5. chair acrobatics | BBI |
| **Calming exercises** | - ...learn to feel their breath in a state of rest.  - ...learn to find peace and relaxation in their bodies by paying attention to their breath.  - ...learn to talk about processes in their body and about their breath.  - ...experience and allow a moment of silence. | 6. sitting still like a frog | E. Snel |
|  |  | 7. with the flow | W. Kinder |
|  |  | 8. mountain and valley breathing (flower breathing) | W. Kinder |
|  |  | 9. breath waves | W. Kinder |
| **Exercises for body awareness** | - ... learn to focus their attention specifically on different parts of their body.  - ... establish a deeper connection to their body and thus get to know it better.  - ... experience a sense of security and centeredness in their body through training their body awareness.  - ... notice an anchoring of attention in the present moment based on their bodily sensations. | 10. foot games | M. Leipold & M. Affeldt |
|  |  | 11. hand awakening | M. Leipold & M. Affeldt |
|  |  | 12. hand awareness | V. Kaltwasser |
|  |  | 13. foot awareness | V. Kaltwasser |
|  |  | 14. mindful listening | D. Recht-schaffen |
| **At the end** | - ... learn about patience, trust, and the ability to let go.  - ... learn to trust in change and not to want to control things that cannot be influenced. | 15. wish tree | E. Snel |

## References

Kaltwasser, V. (2016). *Praxisbuch Achtsamkeit in der Schule: Selbstregulation und Beziehungsfähigkeit als Basis von Bildung*. [*Practice Book Mindfulness in School: Self-Regulation and Relationship Skills as the Basis of Education*]*.* Beltz.

Kinder, W. (2019). *Achtsamkeit. Fantasievolle Übungen, die Kindern Ruhe schenken* [*Mindfulness. Imaginative exercises that give children peace of mind*]*.* Dorling Kindersley.

Leipold, M., & Affeldt, M. (1998). *Kiko – Kinder konzentrieren sich: Klasse 1 - 6 (Dr. A 1.)* [*Kiko - Children concentrate: Class 1 - 6 (Dr. A 1)*]. Schroedel.

Rechtschaffen, D., Harpner, M., & Kabat-Zinn, J. (2016). *Die achtsame Schule: Achtsamkeit als Weg zu mehr Wohlbefinden für Lehrer und Schüler* [*The mindful school: mindfulness as a path to greater well-being for teachers and students*]. Arbor.

Snel, E. (2013). *Stillsitzen wie ein Frosch. Kinderleichte Meditationen für Groß und Klein* (6. Auflage) [*Sitting Still Like a Frog. Easy meditations for young and old* (6th edition)]. Goldmann.
